# Myofunctional Treatment of Anterior Crossbite in a Growing Patient

**DOI:** 10.1155/2020/8899184

**Published:** 2020-10-08

**Authors:** Marianna Pellegrino, Maria Laura Cuzzocrea, Walter Rao, Gioacchino Pellegrino, Sergio Paduano

**Affiliations:** ^1^Independent Researcher, Caserta, Italy; ^2^Independent Researcher, Pavia, Italy; ^3^University of Catanzaro Magna Graecia, Catanzaro, Italy

## Abstract

The purpose of this case report is to add another means of treatment for the anterior crossbite malocclusion in early mixed dentition. The selected functional device is an eruption guidance appliance (EGA). The analysed patient had a functional anterior crossbite, a mandibular protrusion tendency, and a normodivergent growth pattern. The early treatment was suggested to correct the malocclusion and avoid unfavourable occlusal conditions that could end in a class III malocclusion growth pattern. After 18 months of treatment, with night-time use, the malocclusion was completely resolved. This therapy strategy allowed the correction of the sagittal jaws' relationship and maximum control of the vertical dimension. After 2 years of follow-up, the results were preserved. The peculiarity of this kind of intraoral orthodontic tools is the use of the erupting forces rather than the active forces. This early treatment of anterior crossbites with EGA may be considered an effective treatment approach for achieving good functional and aesthetic results.

## 1. Introduction

A dental malocclusion characterized by an increased overjet (>4 mm) has the tendency to improve during the growth, because of the mandibular growth pattern. On the contrary, the percentage of the reversed overjet, indicative of class III malocclusion, tends to increase from the childhood (3%) to the adulthood (5%) [1]. Nonsurgical treatment of class III malocclusion and the anterior crossbite is an orthodontic challenge. A proper diagnosis and an early intervention may be helpful to reduce the worsening of this malocclusion in late adolescence. Many orthopaedic/orthodontic interceptive treatment modalities have been proposed to achieve the class III and the anterior crossbite correction, including the facemask associated with the rapid palatal expander [2], the chin cup [3], the Frankel appliance (FR-3) [4], the bionator, the reverse Twin-block [5], the removable mandibular retractor [6], the double-piece corrector, and the bone anchorage appliances associated to class III elastics [7]. Among these, the reverse-pull headgear is proven to be effective to correct a retrognathic maxilla by many authors. Although there is a moderate amount of evidence about the effectiveness of the facemask appliance in the short term, there is a lack of evidence that the results are maintained in the long term [8].

According to Tollaro et al. [9, 10], the treatment of anterior crossbite and class III malocclusions with a functional appliance in the deciduous dentition produces significant effects on the direction of condylar growth and, consequently, on mandibular size and shape. The functional correction of this malocclusion is achieved using the occlusal forces, which can change the occlusal plane angulation and consequently correct the relationship of the jaws.

The myofunctional intraoral devices which act on the occlusal plane and follow Tollaro's principle can change the inclination of the anterior teeth, reeducate the tongue, reduce the mandibular forward displacement, and improve the chin projection and the soft tissues' harmony [11]. The eruption guidance appliances (EGA) with differential occlusal thickness belong to those functional appliances which can act on the occlusal plane development. According with the current literature, only two studies reported the early treatment of anterior crossbite malocclusion with EGA [12, 13].

The present case report was carried out to investigate the effectiveness of this kind of removable myofunctional appliance to correct the tendency to mandibular protrusion in a growing patient. Very early treatment of functional anterior crossbite can offer the best chance to achieve normal dental and skeletal relationships.

## 2. Case Presentation

The patient was a 6-year-old male with good health status, absence of temporomandibular diseases, any kind of oral habits, familiarity with class III malocclusion (the mother), and good compliance. He was born premature and spent 3 months in the neonatal intensive care unit, where he assumed antibiotics for all the period. He did not do previous orthodontic visits.

### 2.1. Diagnosis

#### 2.1.1. Profile

**Figure 1:**
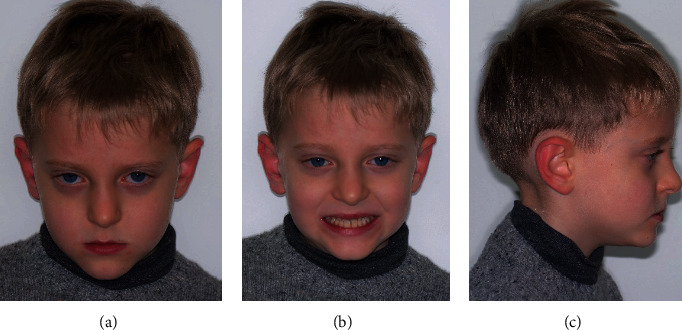
Extraoral pre-treatment pictures: (a) frontal view at rest, (b) frontal view with a smile, and (c) lateral view at rest.

The patient's profile was straight with an open nasolabial angle and a normal labiomental fold. He presented a symmetric face, a slightly increased lower facial third, and a mesocephalic tendency (Figure 1).

#### 2.1.2. Dental Situation

**Figure 2:**
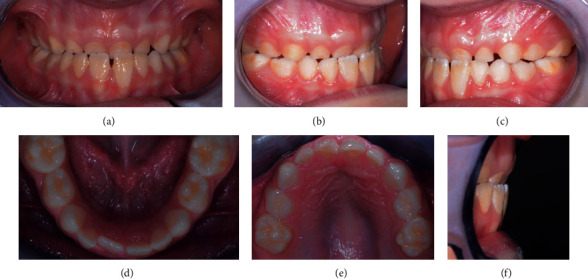
Intraoral pre-treatment pictures: (a) frontal view, (b) frontal view of the right side, (c) lateral view of the left side, (d) occlusal view of the lower arch, (e) occlusal view of the upper arch, and (f) overjet.

His dental situation presented a canine class III and a deciduous molar mesial step on both sides and an anterior crossbite with a reversed overjet (-1.8 mm). The overbite was in the normal range (1.9 mm), and the curve of Spee was flat. On the transversal plane, any kind of malocclusion was detected. On the transversal plane, the only problem to be detected was the maxillary midline deviation to the right (2 mm). The mandibular protrusion was forced by the altered occlusion, and the anterior crossbite was functional because during the mouth opening the midlines are centred. Oral hygiene had to be improved (Figure 2).

#### 2.1.3. Skeletal Situation

**Figure 3:**
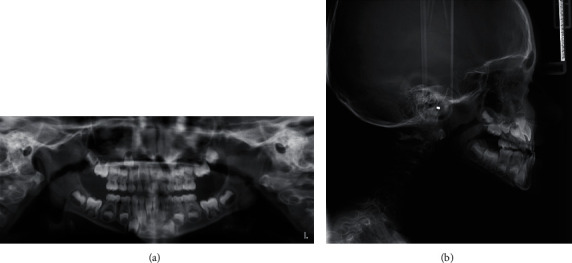
Pre-treatment radiographic records: (a) orthopantomogram and (b) lateral cephalogram.

Lateral cephalogram and orthopantomogram were taken (Figure 3). The patient presented a skeletal class I (ANB = 1.5°) with a protruded mandible (SNB = 84°). He was normodivergent (SnaSnp ^∧^GoMe = 25°; SN ^∧^GoMe = 31°). The interincisal angle was increased (+1 ^∧^ − 1 = 154°) because of the upper incisors' serious retroclination (SnaSnp ^∧^ + 1 = 92°). The lower incisors were normoclined (IMPA = 89°).

The cephalometric values were detected from the lateral cephalogram X-ray. The MBT cephalometric analysis and Jarabak and Fizzel polygon were performed (Table 1). The Jaraback analysis revealed that the patient presented a hypodivergent growth pattern (ArGo ^∧^GoN = 57°; NGo ^∧^GoGn = 73°).

### 2.2. Treatment

The main treatment objectives were to correct the anterior crossbite, to reduce the mandibular protruded growth pattern, to improve the profile, and to change the occlusal plane inclination.

**Figure 4:**
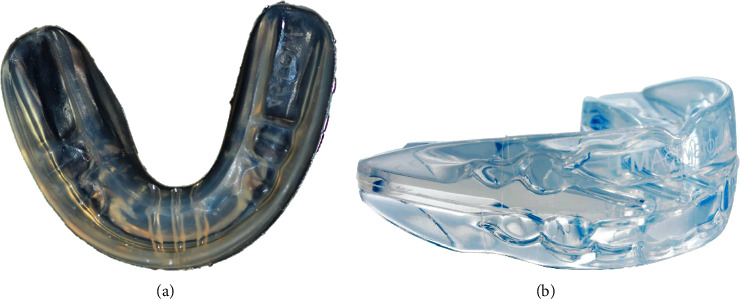
LM-Activator High Short (LM-Instruments Oy, Parainen, Finland): (a) occlusal view and (b) sagittal view.

The orthodontic tool selected to treat this patient was an eruption guidance appliance (EGA). In particular, an LM-Activator High Short size 35 (LM-Instruments Oy, Parainen, Finland) was chosen (Figure 4).

The use of the device was purely nocturnal, and the indication was to use it immediately after dinner (around eight pm) to the following morning. The only exception was the first month; in fact, the EGA was suggested to be worn also 2 hours during the day, to allow the adaptation of the soft tissues and of the perioral muscles. The day-time use was suggested to be associated with emotional and pleasant child activities.

The intraoral appliance size was changed twice with a wider transversal dimension (size 55 and size 60). The progressively increasing appliance size stimulated the maxillary slow expansion and avoided crowding in the upper arch. The intraoral device was always the High version.

The treatment length with the EGA was of 18 months.

### 2.3. Outcome

After the orthodontic phase, the therapy objectives were reached.

#### 2.3.1. Profile

**Figure 5:**
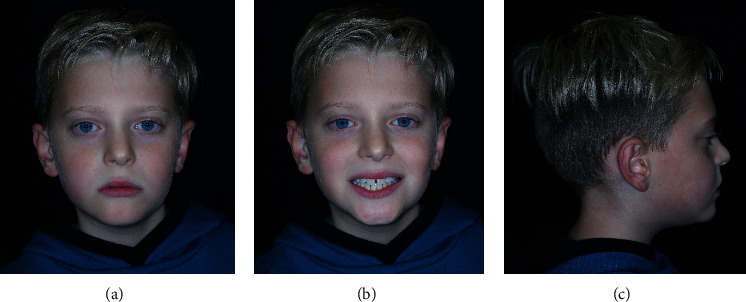
Extraoral post-treatment pictures: (a) frontal view at rest, (b) frontal view with smile, and (c) lateral view at rest.

A more pleasant profile was reached (Figure 5).

#### 2.3.2. Dental Situation

**Figure 6:**
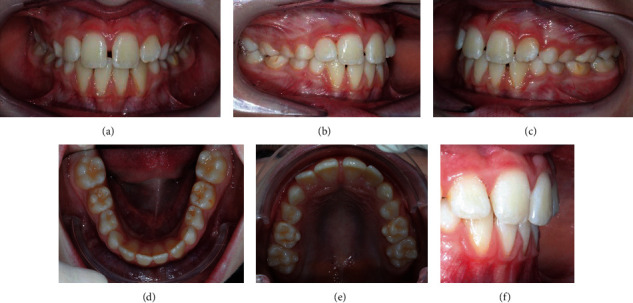
Intraoral post-treatment pictures: (a) frontal view, (b) frontal view of the right side, (c) lateral view of the left side, (d) occlusal view of the lower arch, (e) occlusal view of the upper arch, and (f) overjet.

The anterior crossbite was correct achieving a normal value of overjet (2.7 mm). The upper incisors and the lower incisor were proclined (SnaSnp ^∧^ + 1 = 115°; IMPA = 92°), correcting the interincisal angle (+1 ^∧^ − 1 = 128°), improving the nasolabial angle. The molar mesial step, which could be associated to a class III tendency, was corrected (Figure 6).

#### 2.3.3. Skeletal Situation

**Figure 7:**
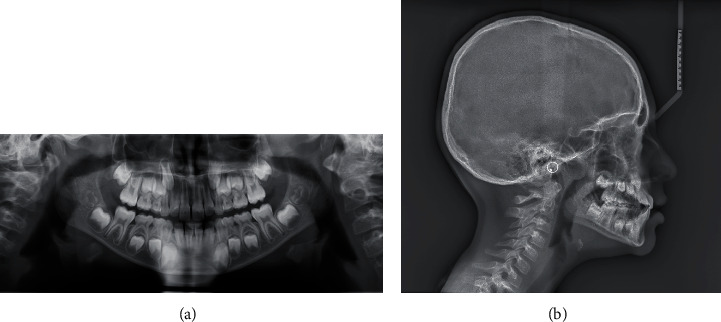
Post-treatment radiographic records: (a) orthopantomogram; and (b) lateral cephalogram.

The sagittal relationship improved (ANB = 2.2°; NA ^∧^APg = 2.2°). The vertical relationship between the mandibular plane and the nasion-sella plane remains the same (SN ^∧^GoMe = 32°) as well as the angle between the mandibular plane and the bispinal plane that increase of 1 degree (SnaSnp ^∧^GoMe = 26°). So, despite a year of growth, the vertical relation remained stable, as well as the Jaraback angles (Table 2). The radiographic records were repeated after the treatment ends (Figure 7).

**Figure 8:**
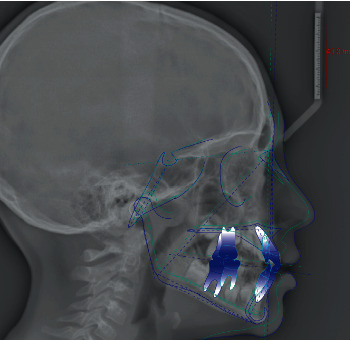
Superimposition: comparison between pre- (green) and post-treatment (blue) cephalograms.

The skeletal, dental, and soft tissue improvements are more evident in the superimposition of the pre- and posttreatment cephalograms (Figure 8).

#### 2.3.4. Follow-Up

**Figure 9:**
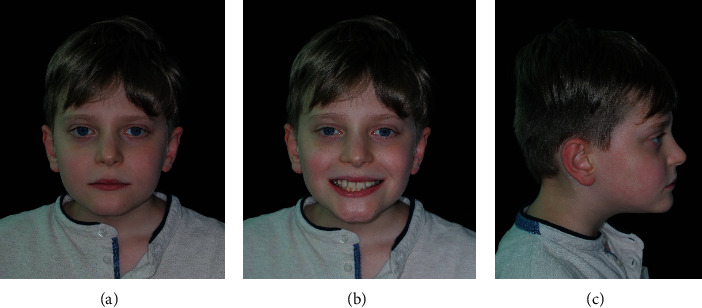
Extraoral follow-up pictures: (a) frontal view at rest, (b) frontal view with a smile, and (c) lateral view at rest.

**Figure 10:**
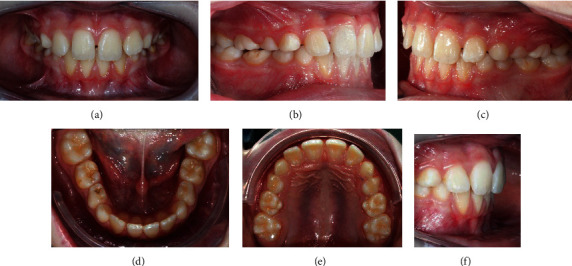
Intraoral follow-up pictures: (a) frontal view, (b) frontal view of the right side, (c) lateral view of the left side, (d) occlusal view of the lower arch, (e) occlusal view of the upper arch, and (f) overjet.

After 2 years from the end of the treatment, the patient still has a straight nice profile and a good vertical proportion is also maintained (Figure 9). He has a dental class I relationship with a proper overjet and overbite (Figure 10). The patient is still using the same type of EGA with a bigger size (65) as active contention during the night.

## 3. Discussion

The functional anterior crossbite should be corrected in early age because of the possible negative influence on the growth pattern. This kind of malocclusion can cause skeletal problem slowing down the maxillary growth and favouring the mandibular forward development [14, 15].

According to Chatzoudi et al.'s meta-analysis, there are lots of appliances available for the treatment of anterior crossbite and class III malocclusion. Among them, the chin cup holds a privileged position as a traditional appliance for the early orthopaedic treatment of this malocclusion. However, the literature reveals controversies and contradictions regarding both its appropriate use and its clinical effectiveness [16].

The intraoral device which has been selected in this case report belongs to the group of eruption guidance appliance (EGA). According to Keski-Nisula et al., the main feature of these devices is that they do not develop active forces to correct teeth position but they use erupting forces, guiding the erupting teeth towards an optimal occlusal position [17]. The presence of different occlusal thickness between the anterior and the posterior area allows a differential eruption of the teeth [18]. The selected EGA (High version) had a major thickness in the molar region to slow down the eruption of the molars and to favour the incisor eruption. In this way, this orthodontic device can create a good interincisal angle and control the dentoalveolar vertical growth. This approach is similar to the SEC appliance, originally presented by Ferro et al. The SEC protocol uses splints, class III elastics, and chin cup to control the vertical dimension. The anterior rotation of the mandible is due to the use of a thinner ramp on the anterior sector associated to vertical elastics [19]. The difference is that the action of the vertical elastics in the SEC protocol is done by the perioral muscles in the EGA approach.

Furthermore, the LM-Activator is similar to a monobloc with the posterior occlusal surface completely flat and the anterior surface with some dental slots. The dental relationship defined by the slots, between the upper and the lower archs, is a canine class I relation. The dental slots have an inclined plane surface, which favours the correction of incisor inclination and dental tipping, following the concept described by Graber et al. [11]. In this way, the lingual inclination of lower incisors, which is a possible side effect of anterior crossbite treatment, is avoided [20].

The correct positioning of dental elements in relation to the apical base and in association to a correct sagittal relation favours a good development of occlusal forces, with a better growth expression [21, 22].

**Figure 11:**
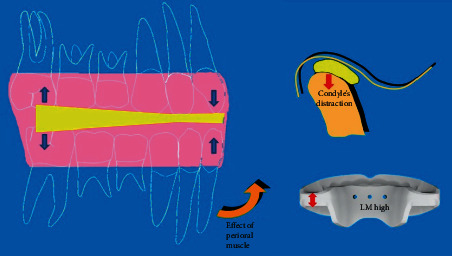
LM-Activator High Action: the increased thickness on the molar section causes the condyle's distraction downward and a counterclockwise rotation of the mandible is due to the perioral muscles.

All these features of the selected EGA are represented in Figure 11. The LM-Activator High allowed a rapid displacement of incisors exploiting the erupting force to control an anterior crossbite, which could have end in a skeletal class III malocclusion.

### 3.1. Limits of the Study

The main limits of this study are the absence of a long-term follow-up and the lack in literature of other studies concerning the treatment of anterior crossbite malocclusion with EGA and its stability during time.

Since the results of this case report are promising, it would be desirable to carry out a clinical study with a longer follow-up, in order to understand the real dentoskeletal effects of this therapeutic approach.

## 4. Conclusion

The night-time use of the selected EGA allowed the anterior crossbite resolution and meanwhile the vertical dimension control. The restoration of a correct sagittal relationship was achieved by exploiting the forces that develop during the occlusion. The malocclusion correction was rapid and effective because of the intervention during early mixed dentition.

## Figures and Tables

**Table 1:** Cephalometric data: pre-treatment.

Parameters	Normal range	Recorded values
SNA	82° ± 2°	84°
SNB	80° ± 2°	84°
ANB	2° ± 2°	1.5°
NA^APg		1.8°
Wits	−1 ± 2 mm	−1.8 mm
SN^GoMe	32° ± 2°	31°
SnaSnp^GoMe	20° ± 5°	25°
SAr^ArGo	143° ± 3°	140°
ArGo^GoN	50° ± 5°	57°
NGo^GoGn	70° ± 5°	73°
+1^SnaSnp	110° ± 6°	92°
IMPA	90° ± 7°	89°
+1^−1	130° ± 5°	154°
Nas^Lab	102° ± 8°	127°

Sna: spina nasalis anterior; Snp: spina nasalis posterior; +1: upper central incisor; -1: lower central incisor; Nas^Lab: naso-labial angle.

**Table 2:** Cephalometric data: post-treatment.

Parameters	Normal range	Recorded values
SNA	82° ± 2°	84°
SNB	80° ± 2°	82°
ANB	2° ± 2°	2.2°
NA^APg		2.2°
Wits	−1 ± 2 mm	−1.8 mm
SN^GoMe	32° ± 2°	32°
SnaSnp^GoMe	20° ± 5°	26°
SAr^ArGo	143° ± 3°	140°
ArGo^GoN	50° ± 5°	55°
NGo^GoGn	70° ± 5°	72°
+1^SnaSnp	110° ± 6°	115°
IMPA	90° ± 7°	92°
+1^−1	130° ± 5°	128°
Nas^Lab	102° ± 8°	110°

Sna: spina nasalis anterior; Snp: spina nasalis posterior; +1: upper central incisor; -1: lower central incisor; Nas^Lab: naso-labial angle.

## Supplementary Materials

Supplementary Materials Care checklist.
